# Vaccinating with a COVID-19 Vaccine: Experience of the Tertiary Allergology Center

**DOI:** 10.15388/Amed.2021.29.1.1

**Published:** 2022-07-26

**Authors:** Eglė Žilėnaitė, Laura Malinauskienė, Kęstutis Černiauskas, Linas Griguola, Kotryna Linauskienė, Violeta Kvedarienė, Anželika Chomičienė

**Affiliations:** Vilnius University, Institute of Clinical Medicine, Clinic of Chest Diseases, Immunology and Allergology, Vilnius, Lithuania, Vilnius University hospital Santaros Klinikos, Pulmonology and allergology department, Santariškių str. 2, Vilnius LT-08661, Lithuania; Vilnius University, Institute of Clinical Medicine, Clinic of Chest Diseases, Immunology and Allergology, Vilnius, Lithuania, Vilnius University hospital Santaros Klinikos, Pulmonology and allergology department, Santariškių str. 2, Vilnius LT-08661, Lithuania; Vilnius University, Institute of Clinical Medicine, Clinic of Chest Diseases, Immunology and Allergology, Vilnius, Lithuania, Vilnius University hospital Santaros Klinikos, Pulmonology and allergology department, Santariškių str. 2, Vilnius LT-08661, Lithuania; Vilnius University, Institute of Clinical Medicine, Clinic of Chest Diseases, Immunology and Allergology, Vilnius, Lithuania, Vilnius University hospital Santaros Klinikos, Pulmonology and allergology department, Santariškių str. 2, Vilnius LT-08661, Lithuania; Vilnius University, Institute of Clinical Medicine, Clinic of Chest Diseases, Immunology and Allergology, Vilnius, Lithuania, Vilnius University hospital Santaros Klinikos, Pulmonology and allergology department, Santariškių str. 2, Vilnius LT-08661, Lithuania; Vilnius University, Institute of Clinical Medicine, Clinic of Chest Diseases, Immunology and Allergology, Vilnius, Lithuania, Vilnius University hospital Santaros Klinikos, Pulmonology and allergology department, Santariškių str. 2, Vilnius LT-08661, Lithuania; Vilnius University, Institute of Clinical Medicine, Clinic of Chest Diseases, Immunology and Allergology, Vilnius, Lithuania, Vilnius University hospital Santaros Klinikos, Pulmonology and allergology department, Santariškių str. 2, Vilnius LT-08661, Lithuania

**Keywords:** COVID-19 vaccination, allergy to vaccines, Pfizer Comirnaty

## Abstract

**Background:**

Allergic reactions after messenger RNA (mRNA)-based COVID-19 vaccines have been reported but detailed descriptions and further actions are not well characterized.

**Objective:**

To describe the symptoms of possible allergic reactions after the mRNA COVID-19 vaccine and outcomes of further vaccination.

**Methods:**

We descriptively analyzed data of adult (≥18 years of age) patients, who were sent for vaccination to our outpatient center for the Diagnostics and Treatment of Allergic and Immune diseases. All patients were vaccinated with the Pfizer–BioNTech Comirnaty® vaccine.

**Results:**

From January 2021 to July 2021 twenty-two patients were vaccinated in our center. Six patients experienced a reaction after the first Comirnaty® dose in different vaccination centers. The majority of them complained of various types of rashes after the first dose, one case was consistent with anaphylaxis. The latter patient was tested with the skin prick using Pfizer–BioNTech Comirnaty® vaccine and the test was negative. Other sixteen patients were vaccinated in our center from the first dose because of past allergic reactions to other medication or due to concomitant mast cell disorder. All patients were vaccinated without any immediate adverse reactions.

**Conclusions:**

None of our patients experienced repeated cutaneous reactions after the second dose. Patients with previous anaphylaxis or mastocytosis also were safely vaccinated.

## Santrauka.

**Įvadas:**

aprašyta alerginių reakcijų į matricines (informacines) RNR (mRNR) vakcinas, tačiau trūksta informacijos, kokia turėtų būti tolesnė vakcinavimo taktika.

**Tikslas:**

aprašyti galimų alerginių reakcijų į mRNR vakcinas simptomus ir pakartotinės vakcinacijos rezultatus.

**Metodai:**

atlikta suaugusių (vyresnių nei 18 metų) pacientų, paskiepytų Vilniaus universiteto ligoninės Santaros klinikų Alerginių ir imuninių ligų diagnostikos ir gydymo dienos stacionare, aprašomoji analizė. Visi pacientai buvo paskiepyti Pfizer-BioNTech Comirnaty® vakcina.

**Rezultatai:**

nuo 2021 metų sausio iki 2021 metų liepos mūsų centre buvo paskiepyti 22 pacientai. Šeši pacientai buvo patyrę reakcijų po pirmos Comirnaty® vakcinos dozės kitame vakcinacijos centre. Daugumai šių žmonių pasireiškė įvairaus tipo bėrimai. Vienam pacientui buvo įtarta anafilaksinė reakcija. Šiam pacientui prieš skiepijimą antra Comirnaty® vakcinos doze buvo atliktas odos dūrio testas su vakcina, testas buvo neigiamas. Kiti 16 pacientų buvo skiepijami Alerginių ir imuninių ligų diagnostikos ir gydymo dienos stacionare nuo pirmos dozės dėl anamnezėje buvusių alerginių reakcijų į kitas vakcinas, vaistus ar dėl esamos putliųjų ląstelių patologijos. Nė vienam iš šių pacientų nepasireiškė greito tipo alerginių reakcijų.

**Išvados:**

nė vienam iš mūsų centre skiepytų pacientų nepasireiškė odos reakcijų po pakartotinės vakcinacijos. Pacientai, kuriems buvo anksčiau diagnozuota putliųjų ląstelių patologija ar anafilaksija, taip pat buvo sėkmingai vakcinuoti.

## Introduction

in March of 2020, World Health Organization assessed that a disease caused by a novel coronavirus (a COVID-19 disease) can be characterized as a pandemic. At remarkable speed, new vaccines were created with the first vaccines being available at the end of December 2020. Currently (at the time of writing this article) registered COVID-19 vaccines in the European Union are: Moderna Spikevax®, AstraZeneca Vaxzevria®, Pfizer–BioNTech Comirnaty® and Jannsen Pharmaceutica NV COVID-19 Vaccine Jansen®. All these vaccines are available in Lithuania.

In general, anaphylactic reactions during vaccination are rare. Although the true incidence of severe allergic reactions is unknown, the rate of immediate hypersensitivity reactions is estimated to be approximately from one per 100,000 to one per 1,000,000 doses [1]. A few studies, analyzing reactions to vaccines, found the anaphylaxis rate to be from 0.65 to 1.53 cases per million vaccine doses [2,3]. However, since the approval of COVID-19 vaccines several cases of anaphylaxis were reported with the calculated rate of the severe allergic reaction being higher than expected (11.1 cases per million doses for Pfizer–BioNTech vaccine) [4]. In response to these reports British Medicines Healthcare Products Regulatory Agency (MHRA) issued stricter recommendations that persons with a history of anaphylaxis to a vaccine, medicine or food should not receive Pfizer–BioNTech vaccine [5]. However, after reviewing the data, MHRA issued new recommendations that contraindications for administering COVID-19 vaccines are a previous severe allergic reaction to the first dose or a known allergy to one of the components of the vaccine [6]. These recommendations are in line with European Medicines Agency, US Food and Drug Administration recommendations.

Given the importance of widespread vaccination, we aimed to review clinical cases of patients vaccinated in our Pulmonology and Allergology center at Vilnius University Hospital Santaros Klinikos and to describe the symptomatology of reactions to the Pfizer vaccine in order to improve future vaccine counseling.

## Methods

We descriptively analyzed data of adult (≥18 years of age) patients, who were sent for vaccination to our Outpatient center for the Diagnostics and Treatment of Allergic and Immune Diseases with a supervision of an allergologist and clinical immunologist. Patients were from the Vilnius region (approx. 1 million inhabitants). Most of the patients were referred by other physicians due to reactions experienced after the first dose or because of concurrent illness which in their opinion could increase the risk of allergic reactions. Due to logistic reasons, all patients were vaccinated with the Pfizer–BioNTech Comirnaty® vaccine.

## Results

From January 2021 to July 2021 twenty-two patients were vaccinated in our center. The majority of patients were women (81.8%), with the median age being 46 years (age range 24–76 years). Six patients experienced a reaction after the first COVID-19 dose in a different vaccination center. Other sixteen patients were vaccinated in our center from the first dose because of past allergic reactions to other medication or had a mast cell disorder. Summary of patient characteristics, previous reactions, and vaccination results is presented in Table 1 and Table 2.

**Table 1.** The summary of patients’ characteristics who were vaccinated with the second dose in the Outpatient center for the Diagnostics and Treatment of Allergic and Immune Diseases.

Patient No	Age	Sex	Vaccine	Reaction after the first dose	Reaction after the second dose
**1.**	68	male	Comirnaty	Rash 24 hours after the first dose. Total rash duration – 8 days. Treated with antihistamines.	No allergic reaction
**2.**	66	female	Comirnaty	Rash, facial swelling, skin itch the second day after vaccination.	No allergic reaction
**3.**	37	female	Comirnaty	Rash, skin itching, fever second day after vaccination. Total rash duration – 4 days. Other: anaphylaxis to paclitaxel.	No allergic reaction
**4.**	69	male	Comirnaty	Red macular rash a few minutes after vaccination. Emergency treatment in vaccination center.	No allergic reaction
**5.**	38	female	Comirnaty	Dizziness, blurry vision a few minutes after vaccination. Loss of consciousness, urine incontinence, seizures. BP 60/40 mmHg. Vomiting 4 hours after vaccination.	Skin prick test with vaccine – negative. No allergic reaction
**6.**	33	female	Comirnaty	Skin rash, arthralgia, general weakness a few minutes after vaccination. Other: low C3. Hemorrhagic vasculitis after MMR vaccine in childhood.	No immediate reaction. Tingling of the tongue, sensation of foreign body in the throat, dyspnea 6 hours after vaccination

**Table 2.** The summary of patients who were vaccinated in Outpatient Center from the first dose.

Patient No	Age	Sex	Vaccine	Reason for vaccinating in allergy day center	Reaction, number of vaccines vaccinated
**1**	38	female	Comirnaty	Anaphylaxis after food ingestion	No reaction, two doses
**2**	55	female	Comirnaty	Probable anaphylaxis after influenza vaccine	No reaction, two doses
**3**	46	female	Comirnaty	Anaphylaxis to paclitaxel	No reaction, two doses
**4**	62	female	Comirnaty	Dyspnea after acetylsalicylic acid. Urticaria, angioedema for 5 years. A drop of blood pressure after dexamethasone injection (for asthma exacerbation)	No reaction, two doses. Second dose in a vaccination center
**5**	76	female	Comirnaty	Probable anaphylaxis during anesthesia	No reaction, two doses
**6**	68	female	Comirnaty	Systemic mastocytosis	No reaction, two doses
**7**	32	female	Comirnaty	Perioperative anaphylaxis (confirmed sensitivity to rocuronium)	No reaction, two doses
**8**	54	male	Comirnaty	Loss of consciousness after DTP vaccine in childhood, treated in ICU for two weeks. Did not get any vaccinations after	No reaction, two doses
**9**	41	female	Comirnaty	Anaphylaxis after Pollinex (ASIT), elevated baseline tryptase, skin rash (probable cutaneous mastocytosis)	No allergic reaction, two doses. General weakness, elevated blood pressure after the second dose
**10**	27	female	Comirnaty	Loss of consciousness after DTP vaccine in childhood, treated with adrenaline, CPR was performed. No vaccinations thereafter	No reaction to the first dose. The second dose recommended in a vaccination center
**11**	30	female	Comirnaty	Rash with vesicles the second day after tick-born encephalitis (TicoVac). Rash duration – 3–4 weeks	No reaction to the first dose. The second dose recommended in a vaccination center
**12**	46	female	Comirnaty	Various reactions to different drugs, chronic urticaria, anxiety.	No reaction to the first dose. The second dose recommended in a vaccination center
**13**	24	female	Comirnaty	Cutaneous mastocytosis	No allergic reaction. Felt facial pruritus, rhinorrhea, pain in the throat after the first dose. Second dose – no reaction
**14**	48	female	Comirnaty	Reactions to B vitamin injections, anxiety	No reaction, two doses
**15**	68	female	Comirnaty	Systemic mastocytosis, systemic reactions to Hymenoptera stings	Two doses, no reaction
**16**	33	male	Comirnaty	Cutaneous mastocytosis	Two doses, no reaction

### Patients with a previous reaction to the COVID-19 vaccine

Three patients experienced an immediate reaction within few minutes after the first vaccination in another vaccination center. Two of those patients experienced rash and general weakness. One patient (patient No 5, Table 1) suffered from an anaphylactic reaction: 3 minutes after the injection she felt dizziness and had a blurry vision. A few minutes later the patient lost consciousness, urinated, and had seizures. Her blood pressure was 60/40 mmHg. She was treated with an intravenous crystalloid solution. Epinephrine was not administered. Four hours later the patient vomited.

Three patients experienced a delayed skin reaction (on the second day after vaccination). All patients complained of skin rash with or without itch, one patient also reported facial swelling. The type of rash was not clarified since the patients were not seen by an allergologist/dermatologist when the rash appeared.

All patients were vaccinated with the second dose of the same vaccine without immediate or delayed-type reaction. For the female patient who experienced a drop of blood pressure (patient No 5, Table 1), a skin prick test with an undiluted vaccine was done before vaccinating with the second dose. The skin prick test was negative.

One patient (patient No 6, Table 1) complained of tingling of the tongue, a sensation of a foreign body in the throat, and dyspnea approximately 6 hours after the second dose. She was examined by an ENT in the Emergency Department. Examination showed a mild swelling of the tongue and no edema of the larynx was noted. Dexamethasone 8 mg and Clemastine 2 mg were administered intramuscularly.

### Patients vaccinated in the Outpatient center for the Diagnostics and Treatment of Allergic and Immune Diseases from the first dose

Ten patients complained of prior reactions to various medications/vaccines, eight of those reactions were immediate. Reported culprit drugs were paclitaxel, rocuronium, acetylsalicylic acid, dexamethasone, B group vitamins, tick-borne encephalitis vaccine, diphtheria/tetanus/pertussis vaccine and influenza vaccine. One patient experienced multiple anaphylactic reactions to various foods. Five patients were vaccinated with the supervision of an allergologist because of underlying systemic or cutaneous mastocytosis, or elevated baseline tryptase level. None of these patients experienced an immediate allergic reaction. One patient (patient No 13, Table 2) felt facial pruritus, rhinorrhea and pain in the throat. She used bilastine 20 mg, mometasone 50 μg nasal spray, and pseudoephedrine/cetirizine 120/5 mg at home. The second dose was administered without complications.

## Discussion

The European Academy of Allergy and Clinical Immunology (EAACI) stresses that allergy to drugs, foods, insect venom, or inhalant allergens, in general, is not a contraindication for any vaccine, including vaccines against COVID-19 disease [6]. The usual observation time after vaccination is 15 min, however, some risk stratification is advisable. EAACI states that previous severe reactions (dyspnea, dizziness, and/or loss of consciousness) after vaccination, drugs, insect stings or food, previous use of epinephrine autoinjector, and use of beta-blockers may impose some risk and, as a safety measure, observation of 30 minutes is recommended for these patients [6]. This statement also recommends that patients with uncontrolled asthma or mast cell disorders should be vaccinated in a hospital setting [6]. Allergy experts of Mass General, Brigham (formerly Partners HealthCare; comprising 16 health care institutions in the New England), and Vanderbilt University Medical Center further clarifies that the history of food, drug(s), venom, or latex allergy except for anaphylaxis, any prior reaction to vaccines, except anaphylaxis, allergic rhinitis, and asthma, poses no additional risk and recommend routine vaccination with 15 minutes observation [7]. Differently from EAACI, Mass General, Brigham, and Vanderbilt allergy experts classified those being diagnosed with mastocytosis and mast cell disorders as a lower risk profile [7].

Regarding cutaneous reactions to COVID-19 vaccines a paper summarizing skin reactions to mRNA vaccines Moderna and Pfizer was published in July 2021 [8]. The authors analyzed 414 cutaneous reactions after vaccination. Most patients with first dose cutaneous reactions did not experience a repeated reaction after the second dose and none of the patients developed severe adverse events neither after the first or the second dose [8]. Authors conclude that cutaneous reactions to COVID-19 vaccines are generally minor and self-limited, and should not discourage vaccination [8]. Our experience with skin reactions is in line with the mentioned article: none of our patients experienced repeated cutaneous reactions after the second dose.

Another issue we encountered was the anxiety of the patients and health care providers regarding the vaccination. The previous statement of the greater risk for allergic patients which was widely escalated by the media and a novelty of the vaccines may be the reason for this anxiety. In some cases, the decision to vaccinate in Allergy Center was influenced by the patient’s fear.

A wide vaccination campaign is the main tool to overcome the COVID-19 pandemic. Clarifying the previous reactions to the vaccine or vaccine components is an important task for an allergologist in this campaign. We suggest not to discourage the patient from further vaccination in case of allergic reaction after the first dose and to refer the patient to an allergologist for further investigations.
